# *In Vivo* Chemical Screening for Modulators of Hematopoiesis and Hematological Diseases

**DOI:** 10.1155/2012/851674

**Published:** 2012-06-19

**Authors:** Yiyun Zhang, J.-R. Joanna Yeh

**Affiliations:** ^1^Cardiovascular Research Center, Massachusetts General Hospital, Charlestown, MA 02129, USA; ^2^Department of Medicine, Harvard Medical School, Boston, MA 02115, USA

## Abstract

*In vivo* chemical screening is a broadly applicable approach not only for dissecting genetic pathways governing hematopoiesis and hematological diseases, but also for finding critical components in those pathways that may be pharmacologically modulated. Both high-throughput chemical screening and facile detection of blood-cell-related phenotypes are feasible in embryonic/larval zebrafish. Two recent studies utilizing phenotypic chemical screens in zebrafish have identified several compounds that promote hematopoietic stem cell formation and reverse the hematopoietic phenotypes of a leukemia oncogene, respectively. These studies illustrate efficient drug discovery processes in zebrafish and reveal novel biological roles of prostaglandin E2 in hematopoietic and leukemia stem cells. Furthermore, the compounds discovered in zebrafish screens have become promising therapeutic candidates against leukemia and included in a clinical trial for enhancing hematopoietic stem cells during hematopoietic cell transplantation.

## 1. Introduction

Zebrafish has been used effectively as a vertebrate model for studying blood cell development and function (for reviews see [1–5]). It is an advantageous model because the optical clarity of its embryos, and their ex utero development enables easy and real-time detection of hematopoietic cells during development. A wide variety of tools and reagents have been developed for *in vivo* labeling and imaging of blood cells and for investigating blood cell function (for reviews of these methods and protocols, see [6–10]). In addition, transient and stable genetic manipulation can link hematopoietic genes to their functions [11–16]. Added to this arsenal of research tools available in zebrafish is *in vivo* chemical screening [17–20]. By exposing zebrafish embryos to a chemical library, bioactive compounds that affect any complex developmental and physiological processes may be identified. Furthermore, *in vivo* chemical screening may be used for uncovering chemical agents that modify a disease phenotype in a whole animal. The compounds that induce a unique biological effect may serve as invaluable probes for identifying critical components of biological pathways, and compounds that can reverse a disease phenotype *in vivo* may have therapeutic potential or shed light on an effective therapeutic target. This innovative approach has created a unique utility for the zebrafish model in chemical biology and contributed to its emerging role in drug discovery (for additional reviews see [21–24]).

## 2. Linking Genes to Their Functions: *In Vivo* Chemical Screens versus Genetic Screens 

Both genetic and *in vivo* chemical screens may be used to dissect genetic pathways that regulate specific biological processes. However, an *in vivo* chemical screen offers the advantage of temporal control that a traditional genetic screen does not. In a genetic screen, gene function is affected from conception. Thus, the role of a gene in early embryonic development may preclude characterization of its roles during later stages. On the other hand, in a chemical screen, compounds that affect the function of a gene can be administered at specific time points and for fixed durations chosen by the investigator so that its roles at different developmental stages may be distinctly determined. In addition, in a genetic screen, the roles of a protein family may sometimes be masked by functional redundancy of its family members. However, chemical modulators may exhibit similar activities against multiple members of a protein family and can, therefore, reveal their *in vivo* cumulative roles. It should be noted that some compounds may affect multiple cellular proteins and thus their on-target effects should be carefully verified using additional chemical agents as well as genetic manipulations. Taken together, *in vivo* chemical screens may complement traditional genetic approaches and uncover hematopoietic genes that cannot be identified in genetic screens.

## 3. Drug Discovery: *In Vivo* Phenotype-Based Chemical Screening versus Target-Based Approach

Currently, the most common approach for identifying potential therapeutics is the target-driven approach (for reviews see [25, 26]). This approach relies on  *a priori* understanding of disease mechanisms to the point of knowing a specific cellular component to be targeted. Thereafter, lead compounds may be obtained using *in vitro* or cell-based assays to determine binding to or modulation of target activity. Typically, these leads will be further optimized using these assays again before being assessed for their *in vivo* efficacy and toxicity. Targets employed by this approach are often enzymes such as kinases that are likely to have small-molecule binding pockets (for more discussions on target druggability, see reviews [26, 27]). Proteins that do not have an obvious pocket, such as transcription factors that often act by recruiting other cofactors, are sometimes dubbed undruggable targets.

Target-based chemical screens performed *in vitro* or in cultured cells are usually very efficient and are able to sample through tens of thousands of compounds. Even so, many drug candidates so identified fail because of poor *in vivo* potency, intolerable side effects, or inability to demonstrate clinical efficacy (for reviews see [28, 29]). In comparison, chemical screens performed in a whole organism may identify working drugs with a higher rate of success since *in vivo *potency and toxicity are evaluated simultaneously during the primary screen [30]. Moreover, by design these screens directly identify compounds that have demonstrated their effectiveness of reversing a disease phenotype *in vivo*. Instead of examining one target as in the target-driven approach, *in vivo* screening is able to interrogate any potential therapeutic targets existing in a biological system that may mediate a disease phenotype, including targets that act in a non-cell-autonomous manner. In many circumstances, the mechanisms of disease pathology are not fully understood, so a target-driven approach is lacking. *In vivo* chemical screening, on the contrary, can be performed before a valid molecular target is identified. 

Although *in vivo* screening has a demonstratedly good likelihood of finding efficacious drug candidates, figuring out their mode of action can be a challenge. A significant amount of effort is usually needed to identify the molecular target of the candidate compound. Nevertheless, due to several important advances in analytical research tools including mass spectrometry, proteomics, genomics, metabolomics, expressional profiling, and chemical informatics as well as novel *in vivo* labeling methods, the efficiency and success rate of target identification have improved significantly in recent years [31–34]. In addition, *in vivo* chemical screens are sometimes performed using chemical libraries consisting of known bioactive compounds, so that the signaling pathways mediating a disease phenotype can be uncovered relatively quickly once chemical suppressors of the phenotype are identified. 

## 4. Efficient *In Vivo* Chemical Screening in Zebrafish 

Some of the model organisms that may be used for *in vivo* chemical screening are *Drosophila*, *C. elegans* and embryonic/larval zebrafish (*Danio rerio*) (for a review see [35]). All of these models have the scalability required for high-throughput screening. Among them, zebrafish is the only vertebrate model and thus possesses the closest physiological similarities to humans.

**Figure 1:**
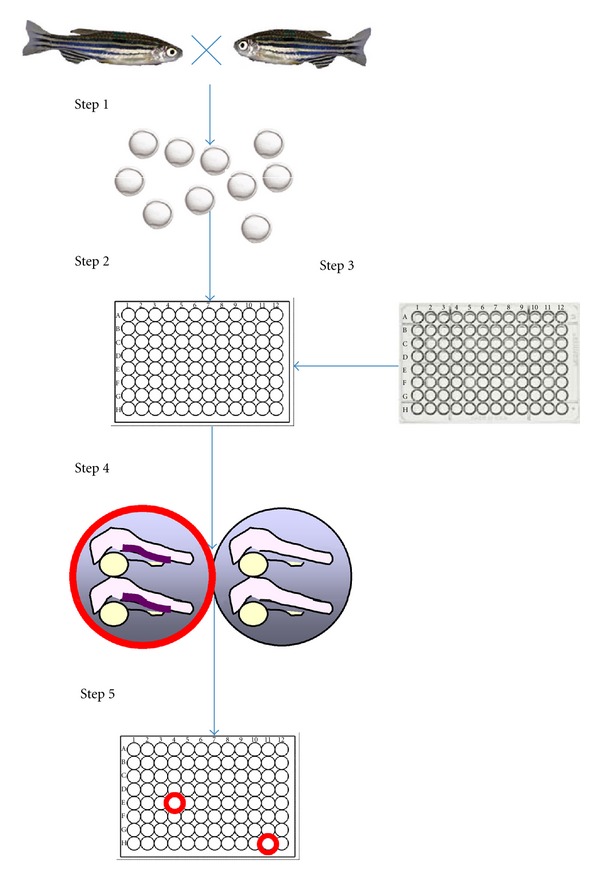
Chemical screening using zebrafish embryos. Step 1—Wild-type, reporter, or mutant zebrafish are crossed to obtain embryos. Step 2—Once reaching an investigator-specified developmental stage (usually between 0–5 days after fertilization), embryos are arrayed into multi-well plates either manually or by automation. Step 3—Compounds from a chemical library are added into the wells containing the embryos using a multichannel pipette or a pin-transfer device. Step 4—After reaching the developmental stage for phenotype manifestation, which is usually within hours to a couple of days after the compound treatment, embryos may be subjected to staining procedures, reporter, or functional assays to detect chemical-induced phenotypes or reversal of genetic phenotypes. The images shown here depict differential hematopoietic gene expression between the compound-treated (red circle) and vehicle-treated (black circle) embryos as detected by RNA *in situ* hybridization. Step 5—*In vivo* phenotypes can be detected by visual inspection or by automated imaging and recording. Thus, the whole screening procedure, once optimized, may be automated for high-throughput experimentation and finished within a few days. In addition, a wide range of phenotypes may be detected *in vivo*.

Features of zebrafish that enable efficient *in vivo* chemical screening are multiple. First is their fecundity. One pair of zebrafish can produce 100–200 embryos each week, so even a medium size aquarium with a couple hundred fish can produce thousands to tens of thousands of embryos per week for screening. Second, zebrafish embryos are small. Generally 3–5 embryos can be arrayed in a well of a 96-well plate containing 100–200 *μ*L of fish water. Further, most cell-permeable small molecules (with octanol:water partition values, or log⁡⁡*P*, above zero) can penetrate zebrafish embryos even when they are inside the chorions [36]. Thus, compounds can be added directly into the water surrounding the embryos. For screens performed in 96-well plates at a 10-*μ*M concentration, only 1-2 micrograms of each compound will be needed for screening. In addition, zebrafish develop quickly, embryos/larvae at 1–5 days after fertilization (dpf) already possess various functional physiological systems. The short developmental timeframe significantly condenses the time needed for experimentation.  Figure 1 shows a schema of *in vivo* chemical screening in zebrafish.

The assays employed for *in vivo* screening will depend on the phenotype of interest. For example, transgenic lines expressing fluorescent proteins under the control of cell-type-specific promoters may be used to track the production or location of specific cell types. Thus, zebrafish *pu.1*, *gata1*, *mpo*, *lyzC*, *csf1r*, *rag2*, *lck*, *CD41,* or *scl *reporter lines among others may be used to identify chemical modulators of myeloid cells, erythrocytes, neutrophils, macrophages, T cells, thrombocytes, or hemangioblasts, respectively [37–45]. Whole-mount immunostaining and RNA *in situ* hybridization may also be used to detect cell proliferation or expression of cell differentiation markers. Even a wide range of physiological outputs and responses may be used as screening readouts, such as chemical-induced enterocolitis, injury-induced inflammation, host-pathogen interactions, and laser-induced thrombosis [46–52]. Some of these assays may be processed by automated liquid handling machines or may be recorded using automated imaging systems and analyzed using customized software [51, 53–56]. Thus, conducting chemical screening in zebrafish provides great potential for identifying modulators of hematopoiesis and hematological diseases.

## 5. Considerations for Screening Designs, Hit Evaluation, and Translation to Humans

### 5.1. Screening Designs

As in any other types of chemical screens, the quality of the hits obtained in zebrafish screens can be directly influenced by the screening designs. For example, if a screen is based on the reduction of the signals in a reporter assay, it may be prone to identifying false positives such as toxic compounds. In this case, a quick visual scan of embryo/larva viability before conducting the reporter assay may help exclude those nonspecific hits. In addition, since proper embryonic development depends on precise execution of multiple sequential processes, compounds added at different times will have the opportunity to affect different developmental steps. Thus, the timing and duration of chemical treatment are also likely to affect the screening outcomes. If a screen utilizes a transgenic line, additional validation steps should be incorporated to examine whether the hit compounds may affect the promoter used for driving the transgene or the stability of the transgene itself. For example, in one of the screens that we have performed, we have identified several hits that suppress the heat shock promoter used for driving the expression of an oncogene rather than the activity of the oncogene [20]. Whenever possible, positive controls should be used to validate that zebrafish models exhibit similar molecular machineries and pharmacological responses as humans do (if the screening purpose is drug discovery) for the biological processes under investigation. This confirmation beforehand will facilitate the likelihood of relevantly translating the findings from zebrafish screens to human conditions.

In addition, it is important to conduct a pilot screen using 100~300 compounds and one screening plate of untreated embryos/larvae to evaluate the robustness and potential variables of the screening methods, including the degrees of natural variations among different clutches of embryos/larvae. A pilot screen may also provide information as regard to the potential hit rates. On one hand, *in vivo* screening methods may cast a broad net for identifying compounds that elicit the phenotype-of-interest through various mechanisms. On the other hand, if the hit rates are higher than 1-2%, researchers may wish to incorporate secondary screening strategies or consider a different screening method in order to limit the hits to the ones that are likely to be of potential interest to the investigators. For example, we previously showed that immediately after the expression of the leukemia oncogene AML1-ETO, *gata1* expression is abolished, whereas *myeloperoxidase* (*mpo*) expression is increased at a later time point [57]. We conducted a chemical suppressor screen and identified various compounds that can restore *gata1 *expression in the presence of AML1-ETO [20]. We have also verified the therapeutic potential of some of the hits identified in this screen, and these results will be discussed in more detail later. Conceivably, a chemical suppressor screen can also be performed based on the reversal of *mpo* upregulation in the same zebrafish model. The latter screening strategy may not only identify compounds that directly antagonize AML1-ETO's effects but also additional compounds that suppress the accumulation of *mpo* ^+^ cells through AML1-ETO-unrelated mechanisms. The choices of screening designs are subject to each investigator's discretion. 

### 5.2. Hit Evaluation and Translation to Humans

The potency, effectiveness, and specificity of the confirmed hits obtained from zebrafish screens have already been demonstrated *in vivo*. Thus, these hits have a high probability of being effective in other *in vivo* systems. Both hematopoietic and other nonhematopoietic effects of these candidate compounds should be evaluated further in embryonic/larval zebrafish. The effects of the candidate compounds on cell differentiation, proliferation, or survival can be evaluated using whole-mount RNA *in situ* hybridization, whole-mount immunostaining or staining with lineage-specific cytological dyes such as Sudan Black for neutrophils and o-dianisidine for hemoglobin. These *in vivo* effects may be assessed facilely using embryonic/larval zebrafish. For example, we have found that AML1-ETO can reprogram hematopoietic cell fate decisions, converting the erythroid cell fate to the granulocytic cell fate. We have also found that nimesulide, a chemical suppressor of AML1-ETO, can reverse these effects in zebrafish. AML1-ETO has been shown to suppress erythroid differentiation in mammalian cells, and we have confirmed that nimesulide can also reverse AML1-ETO's effects in cultured cells [20]. The effects of candidate compounds on leukocyte or thrombocyte function can also be assessed in embryonic/larval zebrafish using an injury model for neutrophil chemotaxis, a bacterial infection model for phagocytosis, or a laser-induced coagulation assay [47, 58, 59]. Moreover, lineage-specific hematopoietic cells can be isolated from control and compound-treated embryos/larvae of various fluorescent reporter lines mentioned earlier by flow cytometry for transcriptional profiling analysis. Interestingly, the nonhematopoietic effects may sometimes provide instrumental information as to the mechanisms of action of the candidate compounds. For example, a candidate compound may cause a developmental phenotype similar to the phenotype caused by other genetic mutations or other chemicals with known bioactivities, suggesting that the candidate compound acts through a similar pathway as these other modulations do. The effects of the candidate compounds can also be evaluated in adult zebrafish using standard hematopoietic assays adapted from mouse models, including irradiation followed by hematopoietic cell transplantation and irradiation recovery assays, as well as leukemic cell xenograft and limiting dilution transplantation [37, 60–64]. The zebrafish provides the investigator the flexibility at which point to verify the effects of these compounds in mammalian systems. While the degree of conservation between zebrafish and mammals in hematopoiesis and in the functions of many hematopoietic cell lineages is high, conservation of humoral regulators and the adaptive immune system is presently less clear. However, rapid advancement in those areas is anticipated. For those biological processes already shown to be highly conserved, the translatability of the screening hits from zebrafish to humans will likely to be high.

## 6. Zebrafish Hematopoiesis and Hematological Disease Models in Zebrafish

### 6.1. Hematopoiesis

Zebrafish possesses a similar set of blood lineages as the mammals [11, 14, 63, 65–71]. The genes involved in blood cell development are also highly conserved between zebrafish and mammals [72, 73]. Thus, it is a suitable model for investigating the genetic pathways regulating hematopoiesis and hematological diseases.

As in mammals, during embryonic development, zebrafish first exhibit a primitive wave of hematopoiesis and later produce several intermediate cell types that eventually contribute to definitive hematopoiesis (for more detailed reviews see [74, 75]). During primitive hematopoiesis, which begins around 11 hours after fertilization (hpf), zebrafish embryos produce myeloid and erythroid cells in two anatomically separate locations. Myeloid cells, which express the transcription factor *pu.1*, are formed in the anterior lateral plate mesoderm (ALM), while erythroid progenitors expressing the *gata1* transcription factor are formed in the posterior lateral plate mesoderm (PLM). It has been shown that hematopoietic cell fate in both blood islands is determined by the expression of these two genes. While knockdown of *pu.1* induces erythropoiesis in the ALM, knockdown of *gata1 *promotes myelopoiesis in the PLM [76, 77]. These results indicate that primitive hematopoiesis in embryonic zebrafish produces bi-potent hematopoietic progenitor cells. Thus, these two synchronously specified blood populations may be useful for identifying important genes that regulate myeloid and erythroid cell fate determination. In a later section of this paper, we will discuss a study that utilizes these cells to uncover some of the AML1-ETO's oncogenic effects that lead to acute myeloid leukemia [20, 57].

In zebrafish, multipotent hematopoietic stem cells (HSCs) originate in the hemogenic endothelium of the aorta, which is equivalent to the aorta-gonad-mesonephros (AGM) in mammals [78]. Using *in vivo* lineage-tracing experiments, it has been shown that these newly emerged HSCs will subsequently colonize a transient hematopoietic tissue called the caudal hematopoietic tissue (CHT), which may be comparable to another mammalian embryonic hematopoietic site in the fetal liver [79–81]. Finally, HSCs from those regions will migrate to and seed both kidney (equivalent to bone marrow in mammals) and thymus, the final hematopoietic organs that remain through adult life [79–81]. As in mammals, zebrafish HSCs express *runx1* and *cmyb*, and *runx1* deficiency abrogates definitive hematopoiesis in fish [78, 82–84]. Several major signaling pathways that regulate HSC formation and homeostasis in mouse models also affect HSC formation in zebrafish, such as the Hedgehog (Hh) pathway and the Notch-Runx pathway [78, 85]. Recently, an *in vivo* chemical screen in zebrafish has identified important roles of the prostaglandin-E2-(PGE2-) Wnt signaling pathway in HSC formation [19, 86], which will be discussed in more detail later. These findings suggest that zebrafish and mammals utilize similar genetic circuitry for regulating HSC formation.

### 6.2. Hematological Disease Models

Due to the easiness of inspecting blood cell phenotypes in zebrafish embryos, a large number of blood mutants have been isolated in three large-scale genetic screens [11, 14, 87, 88]. Many of these blood mutants have defects in the maturation or iron transport of erythrocytes, and their related phenotypes and orthologous gene mutations have been defined in humans [89–91]. Transgenesis approaches have also been used to create various hematological disease models in zebrafish, of which the majority are blood cancer models [20, 38, 57, 92–96]. In these studies, ectopic expression of human oncogenes resulted in zebrafish phenotypes reminiscent of human leukemia characteristics. In addition, investigators can now perform efficient targeted gene disruption in zebrafish using engineered zinc finger nucleases (ZFNs) and transcription activator-like effector (TALE) nucleases [13, 16, 97–100]. In the future, many of these hematological disease models may be used for chemical suppressor screens. The vast array of research tools available in the zebrafish model combined with *in vivo* chemical screening will prove useful in providing novel insights into the molecular mechanisms and potential therapy for hematological diseases.

## 7. *In Vivo* Identification of Hematopoietic Stem Cell (HSC) Chemical Modulators

Compounds that can augment HSC formation and function may exert therapeutic benefits to patients undergoing hematopoietic cell transplantation. North et al. performed a chemical screen to identify small molecules regulating HSC formation in zebrafish embryos [19]. In this study, embryos were exposed between 11 and 36 hpf to 2,357 compounds from three chemical libraries of known bioactive compounds. As mentioned above, HSCs are cmyb^+^ and runx1^+^ and both transcription factors are indispensable for HSC development. By examining *cmyb* and *runx1* expression using RNA *in situ* hybridization, the authors found 35 compounds that increased HSC numbers and another 47 compounds that decreased them. Based on their known bioactivities, they found that 10 of these compounds affect prostanoid biosynthesis. Prostanoids, including prostaglandins, prostacyclins, and thromboxanes, are lipid mediators that play major roles in inflammation and other physiological responses. The cyclooxygenases (COXs), including COX-1 and COX-2 (also known as prostaglandin G/H synthase 1 and 2), convert arachidonic acid into prostaglandin H2, which can then be metabolized into other prostanoids by additional enzymes [101]. Interestingly, the authors found that while exposure to COX inhibitors such as celecoxib and sulindac reduced *cmyb*/*runx1* expression in the hemogenic aorta, exposure to linoleic acid, a precursor of arachidonic acid, enhanced it. Previously it had been shown that prostaglandin E2 (PGE2) is the major prostanoid produced in zebrafish embryos [102]. Thus, North et al. confirmed the involvement of the prostaglandin pathway in HSC formation by incubating zebrafish embryos with PGE2 or selective inhibitors of COX-1 and COX-2, as well as by genetic knockdown of *ptgs1* and *ptgs2* that encode COX-1 and COX-2 proteins, respectively. Subsequently, the authors investigated the expression patterns of *ptgs1* and *ptgs2 *and found that both genes were upregulated at the onset of definitive hematopoiesis. While both genes were expressed in the HSCs, *ptgs1 *was also expressed in the neighboring endothelium. These results strongly suggest that COX-1 and COX-2 promote HSC formation through functions in both the HSCs and their niche. Furthermore, Goessling et al. showed that PGE2 promotes HSC formation by activating the Wnt/*β*-catenin signaling pathway [86].

In their screen, North et al.* also* found 22 compounds that might regulate HSC formation through their effects on blood flow, such as compounds affecting *α*- and *β*-adrenergic receptors, Ca^2+^ or Na^+^/K^+^ channels, nitric oxide (NO) synthesis, or the angiotensin pathway [103]. They showed that blood flow had a positive impact on *cmyb*/*runx1* expression, suggesting that the hemodynamic force on the endothelium might be an inducing factor for the emergence of HSCs. In addition, the authors found that NO donors could stimulate HSC formation even in the* silent heart *mutant, which does not exhibit blood flow. Using mosaic transplantation experiments, they discovered that NO positively regulated HSC through cell-autonomous signaling.

## 8. Validation of HSC Chemical Modulators and Their Clinical Potential

Hematopoietic cell transplantation (HCT) is frequently used in the treatment of hematological malignancies. HSCs not only self-renew but also give rise to all blood lineages and can repopulate an entire hematopoietic system. Patients about to receive HCT need to undergo myeloablation and are treated simultaneously with immunosuppressants to prevent transplant rejection. It is essential that the transplanted HSCs effectively and efficiently engraft in the bone marrow. Various methods aiming to enhance the *in vitro* and *in vivo* expansion of stem/progenitor cells and their homing efficiency to bone marrow are currently under intensive investigation [104–107]. The chemical modulators of HSCs identified by North et al. in zebrafish represent another new therapeutic opportunity.

North et al. showed that *ex vivo* exposure of mouse whole bone marrow (WBM) or purified lin^−^Sca1^+^c-Kit^+^ (LSK) cells to dimethyl-prostaglandin E2 (dmPGE2), a long-lasting derivative of PGE2, significantly increased the progenitor cell numbers as measured by spleen colony-forming units at day 12 after transplantation (CFU-S12) in the recipient mice. Using a limiting dilution competitive repopulation analysis, they found that dmPGE2-treated WBM resulted in 4- and 2.3-fold increases of HSCs in the recipients compared to the untreated cells at 12 and 24 weeks, respectively, following the transplants [19]. To define the mechanisms of action of PGE2, Hoggatt et al.* showed* that *ex vivo* exposure to PGE2 promoted HSC homing efficiency, proliferation, and survival during engraftment [108].

Clinically, sources for HCT include bone marrow, mobilized peripheral blood stem cells (MPBSCs), or human cord blood (hCB). Approximately 20% of HCTs in the United States are conducted using hCB [109]. However, recovery after hCB transplant often takes a very long time due to the limited volume of its source. Thus, Goessling et al. went on to show that dmPGE2 could enhance hCB hematopoietic colony formation *in vitro* and its engraftment in xeno-transplantation [110]. Interestingly, the authors found that hCB samples treated with dmPGE2 exhibited gene expression patterns reminiscent of the HSCs emerged from a vascular niche [110]. Since hCB contains both HSCs and endothelial cells, the authors postulated that dmPGE2 might promote HSC formation from hemogenic endothelial cells, analogous to the scenario in developing zebrafish embryos. Alternatively, Butler et al. have shown that endothelial cells can provide signals for retaining HSC multipotency [111]. Finally, Goessling et al. provided evidence demonstrating preclinical safety of their regimen in nonhuman primate autologous transplantation [110]. Thus, from its initial discovery using an *in vivo* chemical screen in zebrafish, PGE2 is now entering a Phase I clinical trial. 

## 9. *In Vivo* Identification of Acute Myelogenous Leukemia (AML) Chemical Suppressors

### 9.1. AML1-ETO and the t(8;21) AML

Our lab has conducted an *in vivo *chemical screen to identify compounds that could reverse the hematopoietic phenotypes of a human leukemia oncogene [20]. AML1-ETO is a fusion gene resulting from t(8; 21)(q21; q22) chromosomal translocation, and it is one of the most common translocation products in AML. In particular, AML1-ETO expression accounts for 40% of AML in the FAB (French-American-British) M2 subtype [112]. These patients can be characterized by overabundance of granulocytic blast cells.

AML-1, also known as Runx-1, is one of two subunits that form a heterodimeric transcription factor called the core binding factor (CBF). The CBF plays many important roles in hematopoiesis by regulating hematopoietic gene expression (for review see [113]). It has been shown that AML1-ETO exerts a dominant-negative effect on CBF function; however, recent studies also suggest that it produces other gain-of-function effects that account for its oncogenicity [114]. Expression of AML1-ETO enhances HSC expansion both *in vitro* and *in vivo* and promotes myelopoiesis while blocking myeloid maturation [115–119]. Despite intensive studies on gene regulation mediated by AML-ETO, to date no effective therapeutic target has been validated *in vivo*. Thus, we postulated that a phenotype-based, nonbiased approach such as *in vivo* chemical screening might uncover potential therapeutics and identify the critical downstream effectors of AML-ETO.

### 9.2. A Zebrafish Model for AML1-ETO Leukemia

A transgenic zebrafish line Tg(*hsp*:*AML1-ETO*) was established to enable heat-inducible expression of human AML1-ETO [57]. It was found that expression of AML1-ETO in embryonic zebrafish resulted in an accumulation of hematopoietic cells in the posterior blood island [57, 120]. Cytological analysis of the hematopoietic cells isolated from the transgenic embryos showed plentiful immature cells seldom seen in the control samples. In addition, genome-wide expression analysis identified various important similarities between the hematopoietic cells of the transgenic zebrafish and human t(8; 21) leukemia cells [57]. Previously it had been shown that AML1-ETO suppresses erythroid differentiation in human multipotent hematopoietic cells [121]. In the zebrafish model, it was found that AML1-ETO caused the downregulation of *gata1* and the upregulation of *pu.1* in multipotent hematopoietic progenitors, suggesting a conversion of erythroid to myeloid cell fate. Moreover, the accumulated hematopoietic cells strongly expressed the *myeloperoxidase* (*mpo*) gene, indicative of a granulocytic cell fate. A previous study had shown that AML1-ETO downregulates *c/ebp*α**, resulting in a maturation block of the granulocytic cells in human t(8; 21) AML [122]. In the zebrafish model, we also observed a dramatic reduction of *c/ebp*α** expression, suggesting that only two days after its expression in zebrafish embryos, AML1-ETO induced an accumulation of granulocytic blast cells resembling the clinical features of human t(8; 21) AML.

### 9.3. Chemical Screening in the Zebrafish Model of AML-ETO

A library of 2,000 bioactive compounds was screened using the Tg(*hsp*:*AML1-ETO*) zebrafish model [20]. The screening compounds were added to embryos at 12–16 hpf, followed by 1 hour of heat treatment to induce AML1-ETO expression. Fifteen hit compounds were identified by restored *gata1* expression in the transgenic embryos as measured by RNA *in situ *hybridization. We found that some of the compounds affected the heat shock response in zebrafish, preventing AML1-ETO expression. In addition, we identified a histone deacetylase (HDAC) inhibitor sodium valproate as a chemical suppressor of AML1-ETO's effects. HDAC is a transcription corepressor that is known to interact with the ETO moiety of the AML1-ETO protein [123]. It has been shown that recruitment of HDAC is critical for AE's function, and that an HDAC inhibitor trichostatin A (TSA) induces differentiation and apoptosis of a t(8; 21) AML cell line [124]. We have shown previously that TSA also reversed the hematopoietic phenotype of Tg(*hsp*:*AML1-ETO*) zebrafish [57]; therefore, the discovery of sodium valproate validated the biological relevance of the chemical screen performed on the AML1-ETO zebrafish model.

Interestingly, nimesulide, a selective COX-2 inhibitor, was also identified in this screen [20]. Subsequently, we showed that treatments with indomethacin (a nonselective COX inhibitor), NS-398 (a selective COX-2 inhibitor), and nimesulide not only restored *gata1* expression but also inhibited increased expression of *mpo* in the transgenic embryos. Furthermore, we demonstrated that these drugs' effects were on target because they could be reversed by supplementing a downstream metabolite PGE2. Thus, the hematopoietic differentiation defects induced by AML1-ETO *in vivo* can be rescued by inhibiting the COX enzymes.

## 10. Validation of AML Chemical Suppressors and Their Clinical Potential

Since COX inhibitors scored as hits in our screen, we investigated the genes coding for COX proteins and found that *ptgs2* but not *ptgs1* expression was significantly upregulated in the hematopoietic cells of Tg(*hsp*:*AML1-ETO*) zebrafish [20]. At the time of this discovery, very little was known about the potential contribution of the COX enzymes in AML leukemogenesis, although overexpression of COX-2 had been reported in various types of epithelial tumors, including colorectal carcinoma and breast cancers [125, 126]. Moreover, PGE2 had been shown to promote colon cancer cell growth *via* a *β*-catenin-dependent signaling pathway [127, 128]. As in zebrafish, we found that AML1-ETO induced *ptgs2* but not *ptgs1* expression in the K562 human myeloid leukemia cell line [20]. AML1-ETO induced the activity of a *β*-catenin reporter and inhibited erythroid differentiation in these cells, and both effects could be abrogated by NS-398. Subsequently, we found that genetic knockdown of *β*-catenin rescued AML1-ETO's effects in zebrafish embryos [20]. Thus, AML1-ETO affects hematopoietic differentiation through the COX-2/*β*-catenin pathway in both zebrafish and human leukemia cells.

Since the publication of these findings, we have obtained strong evidence indicating that AML1-ETO also signals through a COX-2/*β*-catenin pathway in mouse bone marrow cells (Zhang et al., unpublished results). We have found that COX inhibitors can effectively suppress in vitro serial replating of hematopoietic stem/progenitor cells expressing AML1-ETO as well as AML1-ETO-mediated tumorigenesis in various *in vivo* mouse models (Zhang et al., unpublished results). Two recent studies have also explored the roles of the COX enzymes and *β*-catenin in leukemia stem cells expressing other leukemia oncogenes [129, 130]. In one of the studies, Wang et al. showed that either the MLL-AF9 fusion oncoprotein or coexpression of Hoxa9 and Meis1a could induce *ptgs1* expression and *β*-catenin activation. In addition, inhibiting COX activities using indomethacin attenuated leukemia development induced by MLL-AF9 or by coexpression of Hoxa9 and Meis1a oncogenes [129]. In the other study, Steinert et al. found that a nonselective COX inhibitor sulindac prevented *β*-catenin from being activated and reduced *in vivo* growth of HSCs expressing PML/RAR*α* or PLZF/RAR**α** oncogenes [130].

Collectively, these results suggest that inhibiting the COX enzymes using nonsteroidal anti-inflammatory drugs (NSAIDs) can suppress oncogenic function and *β*-catenin activation in AML leukemia stem cells. Interestingly, case-based studies have also suggested an inverse relationship between NSAID usage and AML incidence [131, 132]. Although PGE2 can induce *β*-catenin expression and augment some aspects of HSC function as discussed above, it has been shown that loss of *β*-catenin does not affect normal hematopoiesis in adult mice [133–136]. At present, a major obstacle for achieving long-term survival of AML patients is relapse. Although chemotherapy can effectively induce remission in the majority of patients, more than 50% of the patients experience relapse within a year after remission [137, 138]. In sum, these results suggest that NSAIDs may impair leukemia stem cell function and thus their clinical efficacy in preventing AML relapse should be explored.

## 11. Final Considerations for Drug Discovery in Zebrafish

In this paper, we presented two specific studies on hematopoiesis that appropriately exemplify the general utility of embryonic zebrafish and phenotypic *in vivo* chemical screening in discovering potential new therapeutics. In these cases, the use of an *in vivo *screening platform allowed the identification of compounds that may act in a noncell autonomous fashion such as hemodynamic forces, bypassed the well-known technical difficulties involved in culturing hematopoietic or leukemia stem cells, and also circumvented the obstacles conferred by undruggable targets or unknown disease mechanisms. Both of the studies uncovered novel biological mechanisms as well as strong candidates for clinical therapeutic use. It is important to note that most of the advantageous features of the zebrafish model occur at its embryonic and larval stages. Thus, a disease phenotype under investigation must manifest during these stages in order to be most effectively exploited for chemical screening. Since multitudinous signaling pathways acting together in zebrafish during early development are also likely to play important roles in maintaining homeostasis in adults and may be disrupted or reactivated during disease progression, a surrogate embryonic phenotype can often be very useful for identifying potential disease modulators. For example, compounds that suppress T-cell development in embryonic zebrafish may demonstrate potent inhibitory effects against T-cell leukemia [18]. Overall, drug discovery in zebrafish benefits from the feasibility of high-throughput chemical screening, closer physiological similarities to human than invertebrate screening strategies, and the ability to create complex disease models not achievable *in vitro*. The possibility of detecting a wider range of hematopoietic phenotypes using innovative assays promises an ever-increasing role for zebrafish in future drug discovery processes.
